# Neuropilin-1 Expression Characterizes T Follicular Helper (Tfh) Cells Activated during B Cell Differentiation in Human Secondary Lymphoid Organs

**DOI:** 10.1371/journal.pone.0085589

**Published:** 2013-12-30

**Authors:** Amédée Renand, Pierre Milpied, Julien Rossignol, Julie Bruneau, François Lemonnier, Michael Dussiot, Séverine Coulon, Olivier Hermine

**Affiliations:** 1 CNRS, UMR 8147, Hôpital Necker, Université Paris Descartes, Sorbonne Paris Cité, Paris, France; 2 Service d’Hématologie Adulte, APHP, Hôpital Necker, Paris, France; 3 Service d’anatomo-pathologie, APHP, Hôpital Necker, Paris, France; 4 Institut Imagine and Université Sorbonne Paris cité, Paris, France; Institut National de la Santé et de la Recherche Médicale, France

## Abstract

T follicular helper (Tfh) cells play an essential role in the development of antigen-specific B cell immunity. Tfh cells regulate the differentiation and survival of activated B cells outside and inside germinal centers (GC) of secondary lymphoid organs. They act through cognate contacts with antigen-presenting B cells, but there is no current marker to specifically identify those Tfh cells which productively interact with B cells. Here we show that neuropilin 1 (Nrp1), a cell surface receptor, is selectively expressed by a subset of Tfh cells in human secondary lymphoid organs. Nrp1 expression on Tfh cells correlates with B cell differentiation *in vivo* and *in vitro*, is transient, and can be induced upon co-culture with autologous memory B cells in a cell contact-dependent manner. Comparative analysis of *ex vivo* Nrp1^+^ and Nrp1^-^ Tfh cells reveals gene expression modulation during activation. Finally, Nrp1 is expressed by malignant Tfh-like cells in a severe case of angioimmunoblastic T-cell lymphoma (AITL) associated with elevated terminal B cell differentiation. Thus, Nrp1 is a specific marker of Tfh cells cognate activation in humans, which may prove useful as a prognostic factor and a therapeutic target in neoplastic diseases associated with Tfh cells activity.

## Introduction

Follicular helper T cells (Tfh) are a specific T cell subset providing help to B cells, thus bolstering the formation of germinal centers (GC), the generation of long-lived plasma cells and of memory B cells. In mouse and human secondary lymphoid organs, Tfh cells are characterized by the expression of CXCR5, the costimulatory molecules ICOS, PD-1 and OX40, and the transcriptional repressor Bcl-6 [1-3]. *In vitro*, these cells are able to induce the production of IgG, IgA and IgM when co-cultured with B cells [4,5]. In addition to the secretion of IL-21 [6], Tfh cells function is dependent on cognate interactions between their TCR and peptide-loaded MHC class II molecules expressed on B cells [7]. The costimulatory molecules ICOS, PD-1, CD40 ligand, and SAP (signaling lymphocytic activation molecule associated protein) play a major role in facilitating the differentiation of B cells [7-10]. Tfh cells differentiation and persistence are dependent on cognate B cell interactions and continued expression of Bcl-6 [11], which can be antagonized by IL-2 signaling and transcriptional repression by Blimp-1 [12,13].

Nrp1 is a receptor of neural guidance cues (class 3 semaphorins) and proangiogenic factors (VEGFs) with crucial roles in the development and function of the nervous and cardiovascular systems [14,15]. In the human immune system, in addition to being a specific marker for plasmacytoid dendritic cells (pDCs) [16], Nrp1 has been attributed a crucial role for the primary activation of T cells by DCs [17]. In mouse, Nrp1 is expressed by recent thymic emigrant invariant NKT cells [18] and is constitutively expressed by natural Foxp3^+^ Treg cells [19-23]. However, in humans Nrp1 expression is rarely found on CD25^+^ Foxp3^+^ Treg cells [24,25]. The majority of T cells expressing Nrp1 *in vivo* do not express CD25, whereas its induction on T cells *in vitro* is dependent on TCR activation, proliferation and expression of CD25 [25].

Tfh cells interact with B cells in secondary lymphoid organs, but there is currently no specific T cell marker for this activity. Although the impact of cognate contacts with Tfh cells on GC B cell differentiation is the focus of intense investigation, little is known of the outcome of such interactions for Tfh cells. TCR engagement on conventional T cells induces the expression of various surface markers such as CD69, CD25 or Nrp1, which are associated with cellular activation and proliferation [25]. Although Tfh cells have little proliferative capacity after TCR stimulation and do not express CD25 during their differentiation induced by dendritic cells [2,26], they strongly express CD69 that would result from multiple contacts with antigen-presenting cells [7]. Additional activation markers that may be specifically induced in Tfh cells after B cell contact are lacking.

Here we characterized Nrp1-expressing T cells in human secondary lymphoid organs. We show that Nrp1 is specifically expressed by a fraction of Tfh cells *in vivo*, that Nrp1 expression on Tfh cells is transient and can be induced on Nrp1^-^ Tfh cells upon contact with autologous memory B cells *in vitro*. Nrp1 induction on Tfh cells is associated with B cell survival and correlates “preferentially” with the percentage of plasmablasts but is not associated to any suppressive activity. We found that Nrp1^-^ and Nrp1^+^ Tfh cells *ex vivo* had similar expression of most Tfh associated genes, yet showed differential expression of certain cytokine and surface receptor genes. Finally, we studied Nrp1 expression by malignant Tfh-like cells in cases of angioimmunoblastic T cell lymphoma (AITL). Our data suggest that Nrp1 expression is specifically induced on Tfh cells after contact with cognate B cells in human and correlate with terminal differentiation of B cells. These findings will help our current understanding of T cell-dependent B cell responses in health and disease.

## Methods

### Human samples

Tonsils were obtained from children undergoing tonsillectomy. Non-malignant lymph nodes (mesenteric, axillary, cervical, submaxillary and mediastinal) were obtained from patients (age range: 2–25 yrs, median: 14 yrs) with non-specific reactive follicular hyperplasia validated by histo-pathological analysis. Briefly, organs were perfused with RPMI 1640 20% FCS, dissociated on a 100 µm nylon membrane, mononuclear cells were obtained after centrifugation over a gradient of Lymphocyte Preparation Medium (PAA) and washed with cold PBS before staining. AITL samples were obtained from cryopreserved lymph node cell suspensions. Participants and next of kin, caretakers, or guardians on the behalf of the minors/children participants provided their written informed consent to participate in this study, which was approved by the Necker Hospital Ethical Committees for human research and were performed according to the European Union guidelines and the declaration of Helsinki.

### Flow cytometry

FITC-labeled anti-CD19 (HIB19), APC-labeled anti-CD38 (HB-7), PE-labeled anti-IgD (IA6-2), FITC-labeled anti-CD69 (FN 50), FITC-labeled anti-CD45RA (5H9), Alexa488-labeled anti-Ki67 (B56), 7AAD (all from BD Biosciences), efluor450-labeled anti-CD3 (OKT3), PE-Cy7-labeled anti-CD4 (RPA-TA), PE or FITC-labeled anti-CD25 (BC96) APC-labeled anti-Foxp3 (236A/E7) (all from eBioscience), FITC-labeled anti-CD57 (HCD57), PERCP-Cy5.5-labeled anti-CXCR5 (TG2/CXCR5), FITC-labeled anti-PD1 (EH12.2H7), Alexa647-labeled anti-ICOS (C398.4A), PERCP-Cy5.5-labeled anti-CCR7 (TG8/CCR7) (all from Biolegend) and PE- or APC-labeled anti-neuropilin1 (anti-BDCA4, 446921, R&D Systems) were used to stain and analyze T cells and B cells populations. Staining was performed as previously described [25]. Data were acquired on a FACSCanto II flow cytometer (BD Biosciences) using FACSDiva software (BD Biosciences) and were analyzed with FlowJo 8.8.2 software (Treestar).

### Cell culture

CD4^+^ T cells were isolated from human tonsils using CD4 microbeads (Miltenyi Biotec). CD4^+^ CD3^+^ CXCR5^-^ Nrp1^-^ (non-Tfh cells), CXCR5^+^ Nrp1^-^ and CXCR5^+^ Nrp1^+^ subsets were isolated by cell sorting. For suppression assays, 100 x 10^3^ non-Tfh cells labeled with 1 µM carboxyfluorescein diacetate succinimidyl ester (CFSE, Invitrogen) were activated with 5 µg/ml anti-CD3ε (UCHT1, R&D Systems) and 2.5 µg/ml anti-CD28 (37407, R&D Systems) plate-bound antibodies in the absence or presence of various ratios of Nrp1^+^ Tfh cells. CD19^+^ B cell subsets were isolated by cell sorting and were cultured (100 x 10^3^ cells per 200 µl per well) alone or with the previously described T cell subsets (50 x 10^3^ cells per 200 µl per well) in 96-well U-bottom tissue culture plates. All cocultures used autologous T and B cells from the same tonsil, unless otherwise stated. For transwell experiments, 24-well U-bottom tissue culture plates and 0.4 µm BD Falcon cell culture inserts (BD Biosciences) were used. B cell viability was assessed by 7-AAD staining after 5 days of culture and live B cells counted with flow cytometry beads (Accucheck, Invitrogen). IgG, IgA and IgM production in the supernatant were determined after 10-14 days of culture by ELISA according to the manufacturer’s protocol (Bethyl). For all experiments, culture medium consisted of RPMI supplemented with 10% FCS, 2mM L-glutamine, 100 U/ml penicillin and 100 mg/ml streptomycin.

### Gene expression analysis

Gene expression analysis was performed on 250 ng mRNA from *ex vivo* isolated Nrp1^-^ Tfh and Nrp1^+^ Tfh cells of 5 distinct tonsils. RNA extraction was performed using RNeasy micro kit (Qiagen). After cDNA synthesis (SuperScript^TM^ III Reverse Transcriptase, Invitrogen), qPCR analysis was performed using custom designed qPCR plates (96 target genes, StellARray, Lonza) and SYBR Premix Ex Taq (Takara), in StepOnePlus Real-time PCR System (Applied Biosystems). Results were analyzed with Microsoft Excel as follows: gene expression (relative to ACTB) was calculated from Ct values with the 2^Ct(ACTB)-Ct(gene)^ formula for each sample independently. For every gene, differential expression in Nrp1^-^ and Nrp1^+^ Tfh cell populations was assessed using a paired student’s t test. All gene expression data (Ct values, gene expression values, fold change and p-value) are compiled in Table S1 available online. 

### Statistical analysis

Statistical analyses were performed using the appropriate tests, as indicated in the figure legends, using Prism 5.0 software (GraphPad) or Microsoft Excel.

## Results

### Nrp1^+^ CD4^+^ T cells in human tonsils are Tfh cells

**Figure 1:**
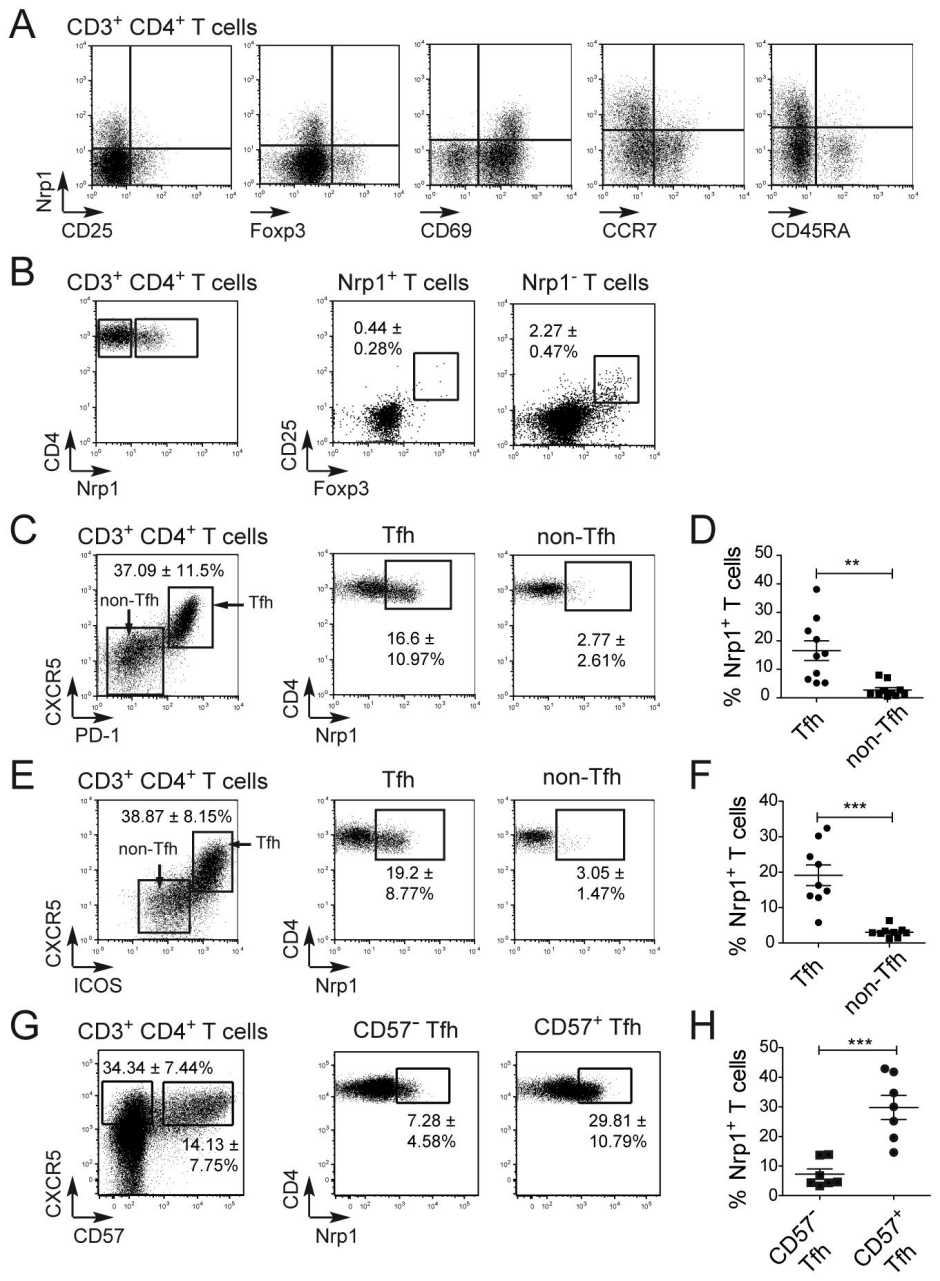
Tonsillar Nrp1^+^ CD4^+^ T cells have a Tfh phenotype *in* *vivo*. (A) Representative flow cytometry analysis of Nrp1 and CD25, Foxp3, CD69, CCR7 and CD45RA co-expression on tonsillar CD3^+^ CD4^+^ T cells population. (B) CD25 and Foxp3 co-expression on tonsillar CD3^+^ CD4^+^ Nrp1^+^ and Nrp1^-^ T cell populations. (C-F) Nrp1 expression on tonsillar Tfh cells and non-Tfh cells, defined as CD3^+^ CD4^+^ CXCR5^+^ PD-1^+^ and CD3^+^ CD4^+^ CXCR5^-^ PD-1^-^ respectively (C-D), or CD3^+^ CD4^+^ CXCR5^+^ ICOS^hi^ and CD3^+^ CD4^+^ CXCR5^-^ ICOS^lo^ respectively (E-F). Numbers in flow cytometry plots indicate the mean percentage ± SD of Tfh cells in CD4^+^ T cells (left) and of Nrp1^+^ cells in Tfh cells (middle) and non-Tfh cells (right) (n=10 tonsils). (G-H) Nrp1 expression on tonsillar CD3^+^ CD4^+^ CXCR5^+^ CD57^+^ and CD3^+^ CD4^+^ CXCR5^-^ CD57^-^ Tfh cells. Data were compared using Student’s impaired t-test (**: p≤0.01, ***: p≤0.001).

In human tonsils, we found Nrp1 to be highly expressed on a subset of CD4^+^ T cells. Tonsillar CD4^+^ T cells expressing Nrp1 had a memory phenotype (CD45RA^-^) and expressed high levels of CD69, but did not express CCR7, excluding their affiliation to the central memory CD4^+^ T cell population (Figure 1 A). We have already demonstrated that, contrary to mice, Nrp1 expression in human is not associated with expression of CD25 and Foxp3 [25]. Here we confirmed that Nrp1 expression was not associated with CD25 or Foxp3 expression (Figure 1 A). Furthermore, very few cells among the CD3^+^ CD4^+^ Nrp1^+^ T cell gate co-expressed CD25 and Foxp3, thereby excluding the affiliation of Nrp1^+^ CD4^+^ T cells to the natural Treg cell population (Figure 1 B), and suggesting that those cells represent a subset of conventional T cells. 

Human tonsils removed by surgery and available for biological studies are chronically inflamed and contain high numbers of Tfh cells. We identified the Tfh cell population in tonsils by CXCR5, ICOS and PD-1 staining on the CD3^+^ CD4^+^ T cell population (Figure 1 C and E). We observed that, among T cells, Nrp1 is almost exclusively expressed by Tfh cells defined as CXCR5^+^ PD-1^+^ (Figure 1 C) or CXCR5^+^ ICOS^hi^ (Figure 1 E and Figure S1), as compared with non-Tfh cells (CXCR5^-^ PD-1^-^ or CXCR5^-^ ICOS^lo^). The difference in Nrp1 expression between PD-1^+^ (Figure 1 D) or ICOS^hi^ (Figure 1 F) Tfh cells and PD-1^-^ or ICOS^lo^ non-Tfh cells was highly significant once data from multiple donors was analyzed. CD57 is a marker of human GC Tfh cells [27]. Nrp1 was expressed in a higher proportion of CD57^+^ Tfh cells than of CD57^-^ Tfh cells (Figure 1 G and H), suggesting Nrp1 is “preferentially” expressed in Tfh cells located in the GC.

**Figure 2:**
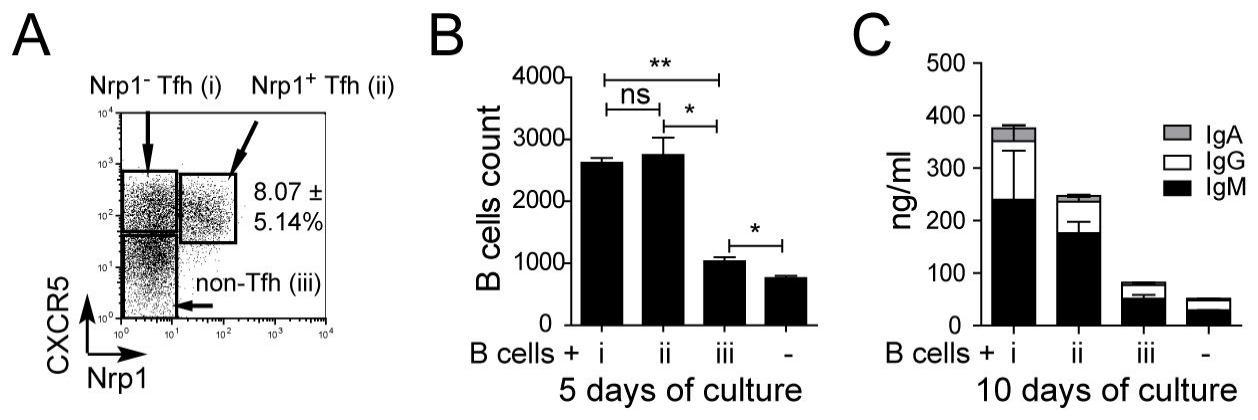
Tonsillar Nrp1^+^ CD4^+^ T cells support survival and Ig production of B cells *in* *vitro*. (A) Flow cytometry analysis of Nrp1^-^ Tfh (CXCR5^+^ Nrp1^-^: i), Nrp1^+^ Tfh (CXCR5^+^ Nrp1^+^: ii) and non-Tfh cells (CXCR5^-^ Nrp1^-^: iii). (B-C) These three T cell subsets were cultured with B cells without exogenous stimulation, and compared for their ability to maintain B-cell survival after 5 days (B) and to induce the production of IgG, IgA and IgM (C) after 10 days of culture. Representative data from one out of four experiments are shown. Data were compared using Student’s impaired t-test (ns: not significant, *: p≤0.05, **: p≤0.01).

In order to confirm that Nrp1^+^ T cells belonged to the Tfh cell population, we tested their ability to induce B cell survival and immunoglobulin production *in vitro*. For this, we sorted different tonsillar CD4^+^ T cell populations based on CXCR5 and Nrp1 expression (Figure 2 A), and used them in coculture assays with autologous B cells in the absence of exogenous stimulation. We observed that, unlike non-Tfh cells, Nrp1^+^ Tfh cells had the ability to sustain B cell survival (Figure 2 B) and production of IgM, IgG and IgA (Figure 2 C). Interestingly, although Nrp1^+^ Tfh cells were as potent as their Nrp1^-^ counterparts for supporting B cell survival, Nrp1^+^ Tfh cells displayed a slightly lower capacity to induce immunoglobulin production than Nrp1^-^ Tfh cells. Altogether, these results show that Nrp1^+^ CD4^+^ T cells in human tonsils are Tfh cells.

### Nrp1^+^ CD4^+^ T cells in human tonsils are not proliferating and have no suppressive activity

**Figure 3:**
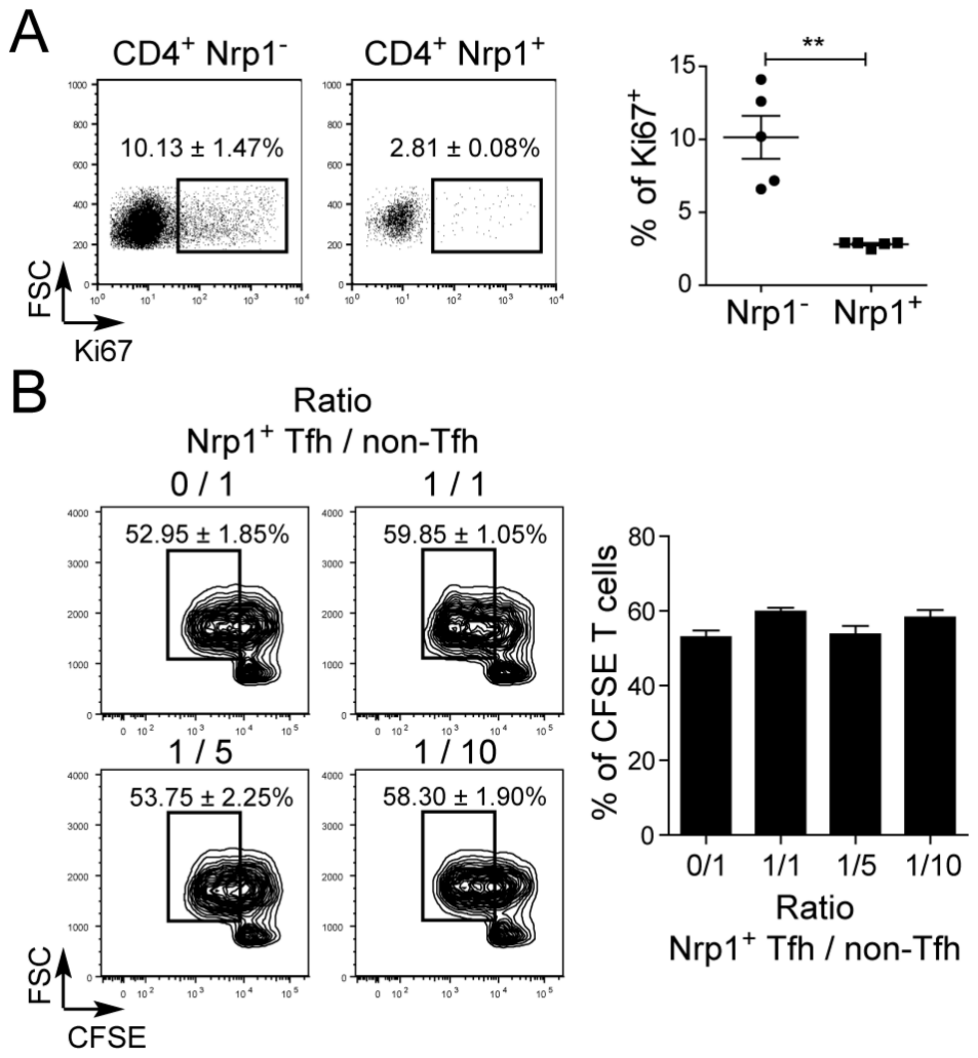
Nrp1^+^ T cells are not proliferating and have no regulatory activity. (A) Ki67 expression in tonsillar CD3^+^ CD4^+^ Nrp1^-^ and Nrp1^+^ T cells. Five tonsils were analyzed and the expression data was compared with a Student’s paired t-test (**: p≤0.01). (B) The regulatory function of Nrp1^+^ Tfh cells was tested in coculture experiments with CFSE-labeled non-Tfh cells as described in Materials and Methods. The percentage of non-Tfh cells having diluted CFSE after 5 days of culture with various ratios of Nrp1^+^ Tfh cells was analyzed by flow cytometry. Data pooled from three distinct experiments are summarized here.

In previous studies in mice and humans, the expression of Nrp1 in T cells has been associated with either proliferation or suppressive activity [18-25]. We therefore investigated whether Nrp1^+^ CD4^+^ T cells may correspond to a subset of Tfh cells in proliferation or to the recently described Foxp3^+^ regulatory Tfh cells [28,29]. In tonsils, Nrp1^+^ Tfh cells did not express the proliferation associated marker Ki67, suggesting that these cells are quiescent *in vivo* (Figure 3 A). Moreover, Nrp1^+^ Tfh cells did not express the transcription factor Foxp3 (Figure 1 A and B)[25], and did not suppress the proliferation of non-Tfh cells in an *in vitro* coculture assay, at the difference of CD25^hi^ CD4^+^ peripheral blood T cells (Figure 3 B and data not show). 

### Nrp1 expression correlates with B cell differentiation in secondary lymphoid organs

**Figure 4:**
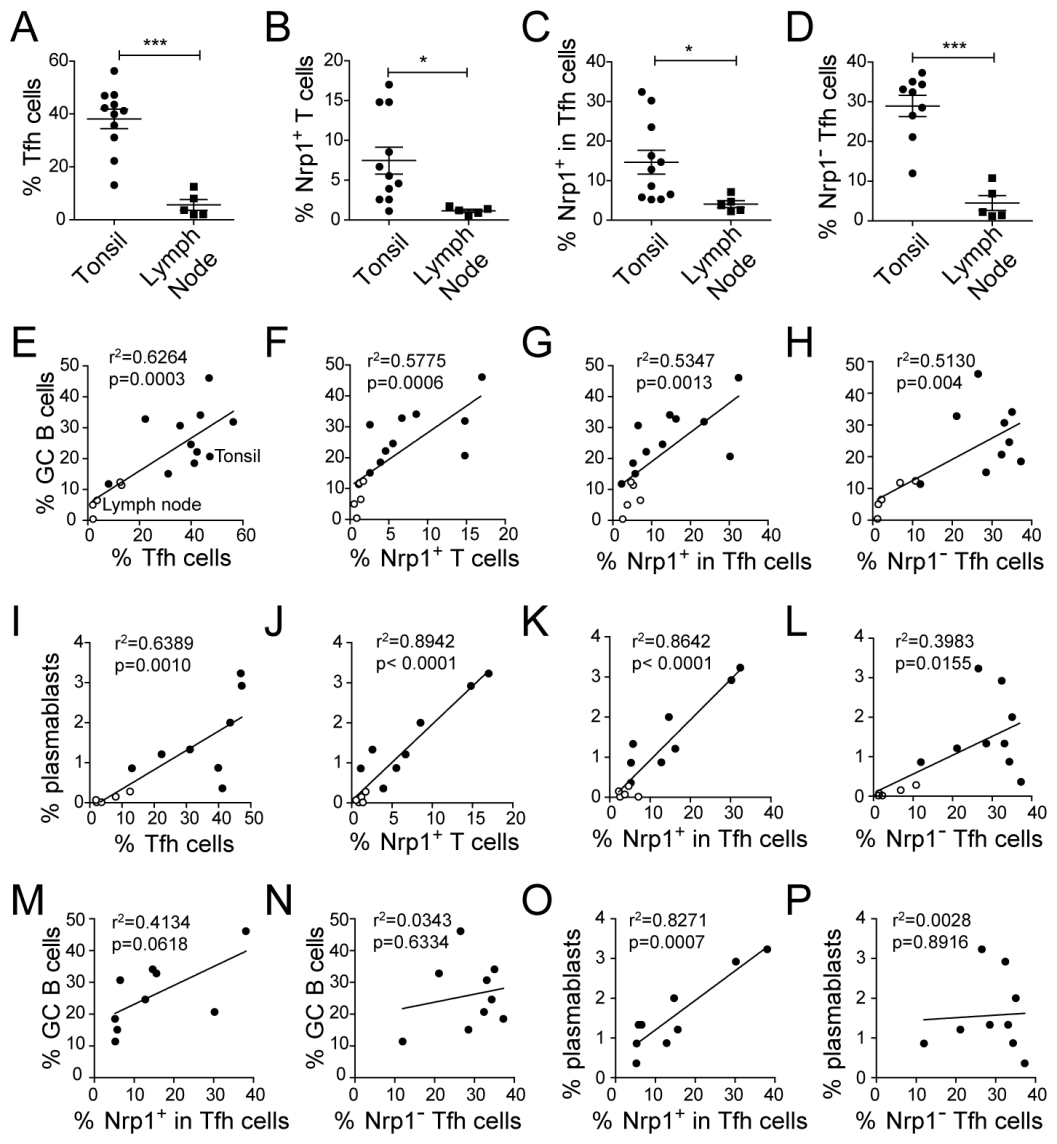
Correlation between Nrp1 expression and germinal center activity in secondary lymphoid organs. Tonsils and non-malignant reactive lymph nodes were compared for their percentages of Tfh cells (A), Nrp1^+^ T cells (B) and Nrp1^-^ Tfh cells (D) in CD4^+^ T cells and for their percentage of Nrp1^+^ cells in Tfh cells (C) . (E-L) Correlation between the percentage of Tfh cells (E and I), Nrp1^+^ T cells (F and J), Nrp1^+^ cells in Tfh cells (G and K), and Nrp1^-^ Tfh cells (H and L) and the percentage of GC B cells (E-H) or plasmablasts (I-L) among CD19^+^ cells in tonsils (full circle) and non-malignant reactive lymph nodes (white circle). (M-P) Correlation between the percentage of Nrp1^+^ cells in Tfh cells (M and O), or the percentage of Nrp1^-^ Tfh cells (N and P), and the percentage of GC B cells (M-N) or plasmablasts (O-P) among CD19^+^ cells only in tonsils only. Data were compared using Student’s impaired t-test (*: p≤0.05, ***: p≤0.001) (A-D). For correlation analyses, the correlation coefficient r^2^ and the associated p-value are shown.

To understand the significance of *in vivo* Nrp1 expression, we compared T and B cell populations in human tonsils and non-malignant reactive lymph nodes. Tonsils contained significantly more total Tfh cells (CXCR5^+^ PD-1^+^) and Nrp1^-^ Tfh cells than reactive lymph nodes (Figure 4 A and D). The proportion of Nrp1^+^ T cells was also higher in tonsils on total CD4^+^ T cells (Figure 4 B) or in Tfh cells (Figure 4 C). When we analyzed the distribution of B cell subpopulations defined by the CD38 and IgD markers (Figure S1) [30,31], we found that tonsils contained a significantly higher proportion of IgD^-^ CD38^+^ GC B cells (26 % vs 7.24%) and IgD^-^ CD38^high^ plasmablasts (1.6% vs 0.1%). However percentages of total CD4^+^ and CD19^+^ cells were not different between tonsils and reactive lymph nodes (data not show). As expected, the percentage of GC B cells in all lymph nodes and tonsils was correlated with total Tfh cells (Figure 4 E); it was also correlated with Nrp1-expressing CD4^+^ T cells (Figure 4 F), the percentage of Nrp1^+^ cells within Tfh cells (Figure 4 G) and with Nrp1^-^ Tfh cells (Figure 4 H). Interestingly, the correlation between plasmablasts and Nrp1^+^ T cells (or the percentage of Nrp1^+^ cells within Tfh cells) was much tighter and more significant compared to the correlation with total Tfh cells or with Nrp1^-^ Tfh cells (Figure 4 I-L). 

Because germinal center activity and terminal differentiation were more important in tonsils in contrast to lymph nodes, we also analyzed the significance of Nrp1 expression only in tonsils (Figure 4 M-P). The percentage of Nrp1^+^ cells within Tfh cells was loosely correlated to GC B cells, but very tightly correlated to plasmablasts in tonsils (Figure 4 M and O). By contrast, Nrp1^-^ Tfh cells did not correlate with GC B cells or plasmablasts when only tonsils were analyzed. Altogether, these results suggest that Nrp1 expression is strongly associated with Tfh cells activity involved in the regulation of B cell differentiation *in situ*.

### Nrp1 expression by Tfh cells is dependent on cognate B cell contacts and correlates with B cell differentiation

**Figure 5:**
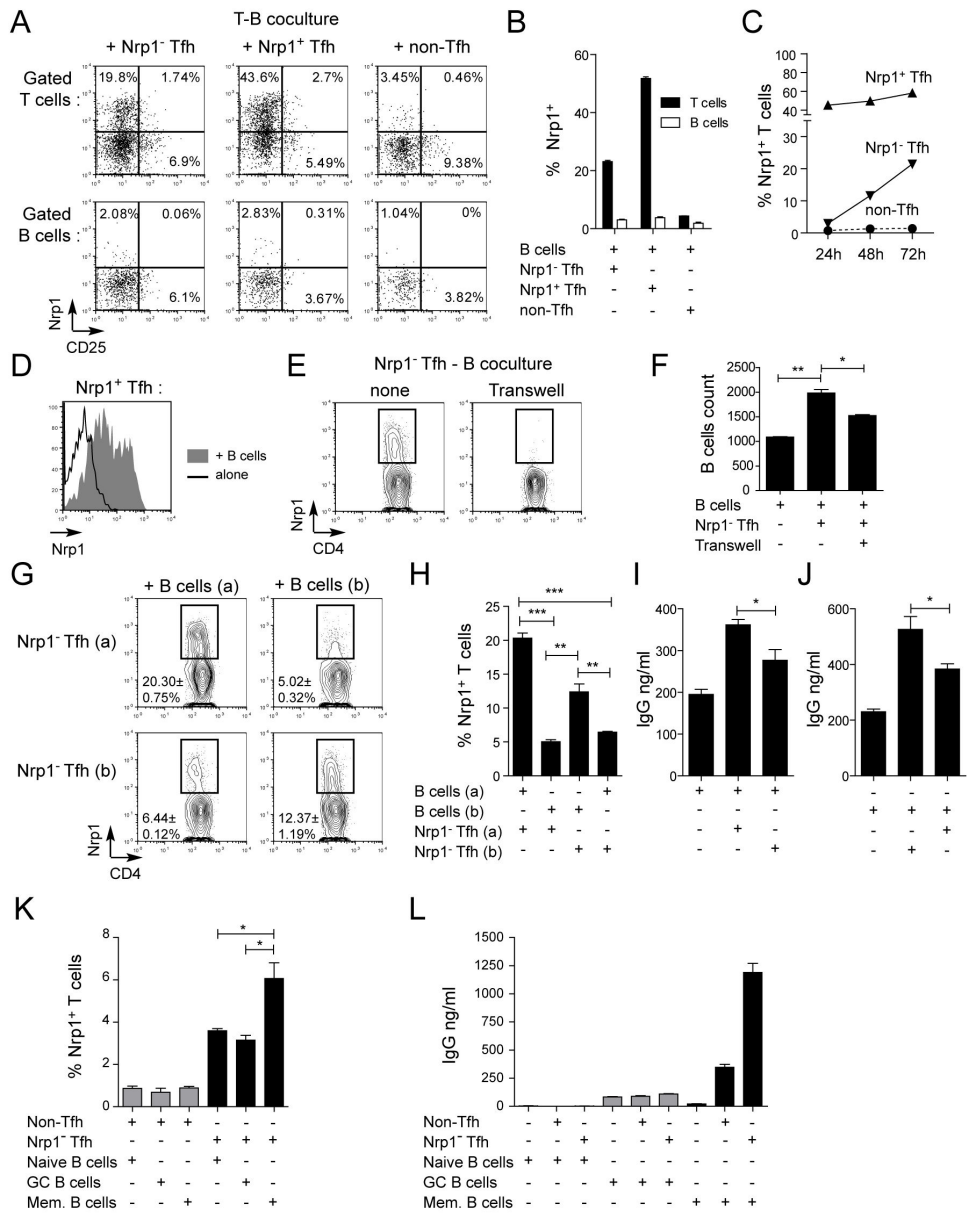
Nrp1 expression by Tfh cells requires cognate B cell contact and reflects Tfh activity *in* *vitro*. (A-C) Nrp1^-^ Tfh, Nrp1^+^ Tfh and non-Tfh cells were sorted as in Figure 1 and cultured with autologous B cells in the absence of exogenous stimuli. (A) Representative expression of CD25 and Nrp1 on T cells (top) and B cells (bottom) after 5 days of culture. (B) Percentage of Nrp1^+^ cells among T or B cells after 5 days of culture. (C) Percentage of Nrp1^+^ cells among T cells after 24, 48 and 72 hours of culture. In B and C, representative data from one out of three distinct experiments are shown. (D) Nrp1 expression on sorted Nrp1^+^ Tfh cells after 5 days of culture alone (dark line) or with autologous B cells (shaded histogram). (E-F) Nrp1^-^ Tfh cells were cultured with autologous B cells in the absence or presence of a transwell membrane separating the two cell types. (E) Representative Nrp1 expression on T cells after 5 days of culture. (F) Number of live B cells per well after 5 days of culture. (G-J) Autologous or allogeneic cocultures were performed using Nrp1^-^ Tfh and B cells sorted from two distinct tonsils (a) and (b). (G) Nrp1 expression on T cells after 5 days of culture. Data represent one experiment with triplicate wells and are representative of three distinct experiments. (I-J) IgG production in culture supernatant after 10 days of culture. (K-L) Sorted naive, GC or memory B cells were cultured in the absence or presence of autologous CXCR5^-^ Nrp1^-^ non-Tfh cells or CXCR5^+^ Nrp1^-^ Tfh cells. (K) Nrp1 expression on non-Tfh cells and Nrp1^-^ Tfh cells after 4 days of culture. (L) IgG production in culture supernatants after 14 days of culture. Data were compared using Student’s impaired t-test (*: p≤0.05, **: p≤0.01).

Next, we sought to determine the origin of Nrp1 expression on Tfh cells and the significance of this expression. When cultured in the presence of B cells, Nrp1^-^ Tfh cells upregulated Nrp1 in the absence of any other stimulation (Figure 5 A and B). As suggested by the absence of CD25 expression on human Nrp1^+^ T cells *in vivo* [25,32], Nrp1 expression in Tfh cells cocultured with B cells was not associated with CD25 upregulation. Nrp1 upregulation was specific to Tfh cells, as it was not observed in non-Tfh cells or B cells in these experiments (Figure 5 A and B). Nrp1 upregulation in Nrp1^-^ Tfh cells started after 24h of culture with B cells and increased with time (Figure 5 C). When cultured alone, Nrp1^-^ Tfh cells did not spontaneously express Nrp1 (data not shown). Nrp1^+^ Tfh cells lost Nrp1 expression when cultured in the absence of B cells, but most remained Nrp1^+^ when cocultured with B cells (Figure 5 D). Transwell experiments showed that cell contact was required for Nrp1 upregulation in Tfh cells and for their pro-survival effect on B cells (Figure 5 E and F). Circulating CXCR5^+^ Tfh cells, which have Tfh function *ex vivo* [33] but do not interact with B cells in the blood, did not express Nrp1 (Figure S2), further suggesting that Nrp1 expression on Tfh cells is lost shortly after cessation of Tfh – B cell contacts. 

Tfh cell regulation of B cell differentiation and survival depends on cognate peptide-MHCII / TCR interactions. Next we investigated whether Nrp1 expression in Tfh cells was dependent on such antigen-restricted interactions with B cells. We reasoned that coculturing Tfh cells and B cells from the same tonsil favored interaction of cells with the same foreign antigen specificity, whereas taking cells from different donors likely supported only non antigen-specific interactions and allogeneic activation. High expression of Nrp1 in Nrp1^-^ Tfh cells was observed after autologous, but not allogeneic, coculture experiments (Figure 5 G and H). This expression was associated with significantly higher IgG production during autologous responses (Figure 5 I and J). Reduced, yet reproductive, induction of Nrp1 in Tfh cells in allogeneic cocultures likely reflected non antigen-restricted allogeneic activation. Altogether, these experiments show that Nrp1 expression is specific of Tfh cells and marks their cognate engagement with B cells.

Thus, Nrp1 expression characterized Tfh cells engaging cognate B cells and correlated with antibody production (reflecting terminal differentiation). Next, we sought to determine which B cell subset was best able to induce Nrp1 on Tfh cells *in vitro*. Tonsillar B cell subsets (naive, GC and memory) were sorted based on expression of CD38 and IgD (Figure S1) and cultured with CXCR5^+^ Nrp1^-^ Tfh cells or CXCR5^-^ Nrp1^-^ non-Tfh cells, and Nrp1 induction and IgG production were measured after 4 and 14 days, respectively (Figure 5 K and L). These experiments confirmed that non-Tfh cells (including naive and memory T cells) had very limited capacity to induce B cells differentiation *in vitro* and could not upregulate Nrp1 expression. Interestingly, we found that memory B cells were the only B cell subset able to induce significant Nrp1 expression in Nrp1^-^ Tfh cells, and Nrp1 induction on Tfh was associated with strong IgG production in these co-cultures. These experiments, performed in the absence of exogenous stimulation, show that Nrp1 is specifically induced on Tfh cells upon activation of memory B cell terminal differentiation *in vitro*. 

### Gene expression profiles of Nrp1^-^ and Nrp1^+^ Tfh cells

**Figure 6:**
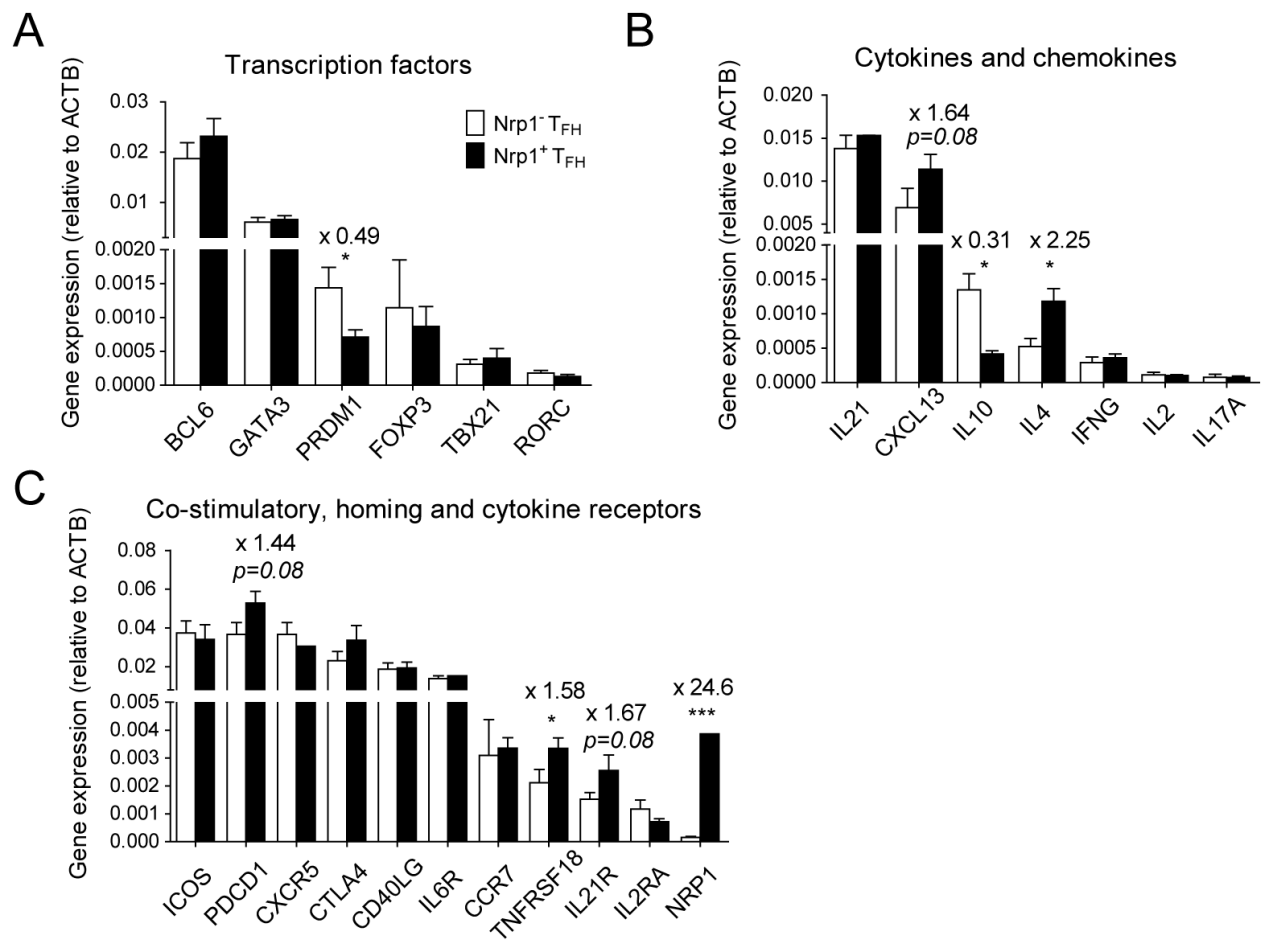
Gene expression profiles of Nrp1^-^ and Nrp1^+^ Tfh cells. Nrp1^-^ and Nrp1^+^ Tfh cells were sorted from 5 distinct tonsils and analyzed for gene expression as described in Materials and Methods. Gene expression (normalized to ACTB) of a selection of relevant transcription factors (A), cytokines and chemokines (B), co-stimulatory, homing and cytokine receptors (C) in Nrp1^-^ and Nrp1^+^ Tfh cells is shown here. Numbers above bars indicate fold change and p-value (paired student’s t test).

Our correlation studies and *in vitro* experiments strongly suggested that Nrp1 expression in tonsillar Tfh cells *in vivo* marked cells that had recently interacted with cognate antigen-presenting B cells and mediating their differentiation. We analyzed the expression of several immunologically relevant genes in *ex vivo* purified tonsillar Nrp1^-^ and Nrp1^+^ Tfh cells to identify key molecular differences between the two subsets (Figure 6 and Table S1). Consistent with both subsets being CXCR5^+^ Tfh cells, Nrp1^-^ and Nrp1^+^ Tfh cells showed high expression of the Tfh-associated genes *BCL6*, *IL21*, *CXCL13*, *ICOS*, *PDCD1* (encoding PD-1), *CXCR5*, *CD40LG* (encoding CD40L) and *IL6R*, but low expression of genes associated with other T helper lineages like *FOXP3*, *TBX21* (encoding T-bet), *RORC* (encoding ROR-γt), *IFNG* (encoding IFN-γ), *IL2*, *IL17A*, *CCR7* and *IL2RA* (encoding CD25) (Figure 6 A-C). Moreover, *NRP1* expression was restricted to Nrp1^+^ Tfh cells, thus further confirming the purity of the sorted cell populations (Figure 6 C).

Nrp1^+^ Tfh cells differed from their Nrp1^-^ counterparts by expressing significantly less *PRDM1* (encoding Blimp-1) and *IL10*, but more *IL4* and *TNFSFR18* (encoding GITR) (Figure 6 A-C). Nrp1^+^ Tfh cells also expressed more *CXCL13*, more *PDCD1*, and more *IL21R* than Nrp1^-^ Tfh cells, although these differences did not reach statistical significance (Figure 6 B-C). There was also a significant 2-fold increase in the expression of *ROCK2*, *VEGFA* and *STAT4* in Nrp1^+^ Tfh cells (Table S1). These results suggest that cognate contact with B cells *in vivo* imprints a distinct molecular program in human Tfh cells that goes beyond the upregulation of Nrp1 and likely serves a function in B cell differentiation and the GC reaction. 

### Nrp1 expression in malignant PD-1^+^ Tfh-like cells of angioimmunoblastic T-cell lymphoma (AITL)

**Figure 7:**
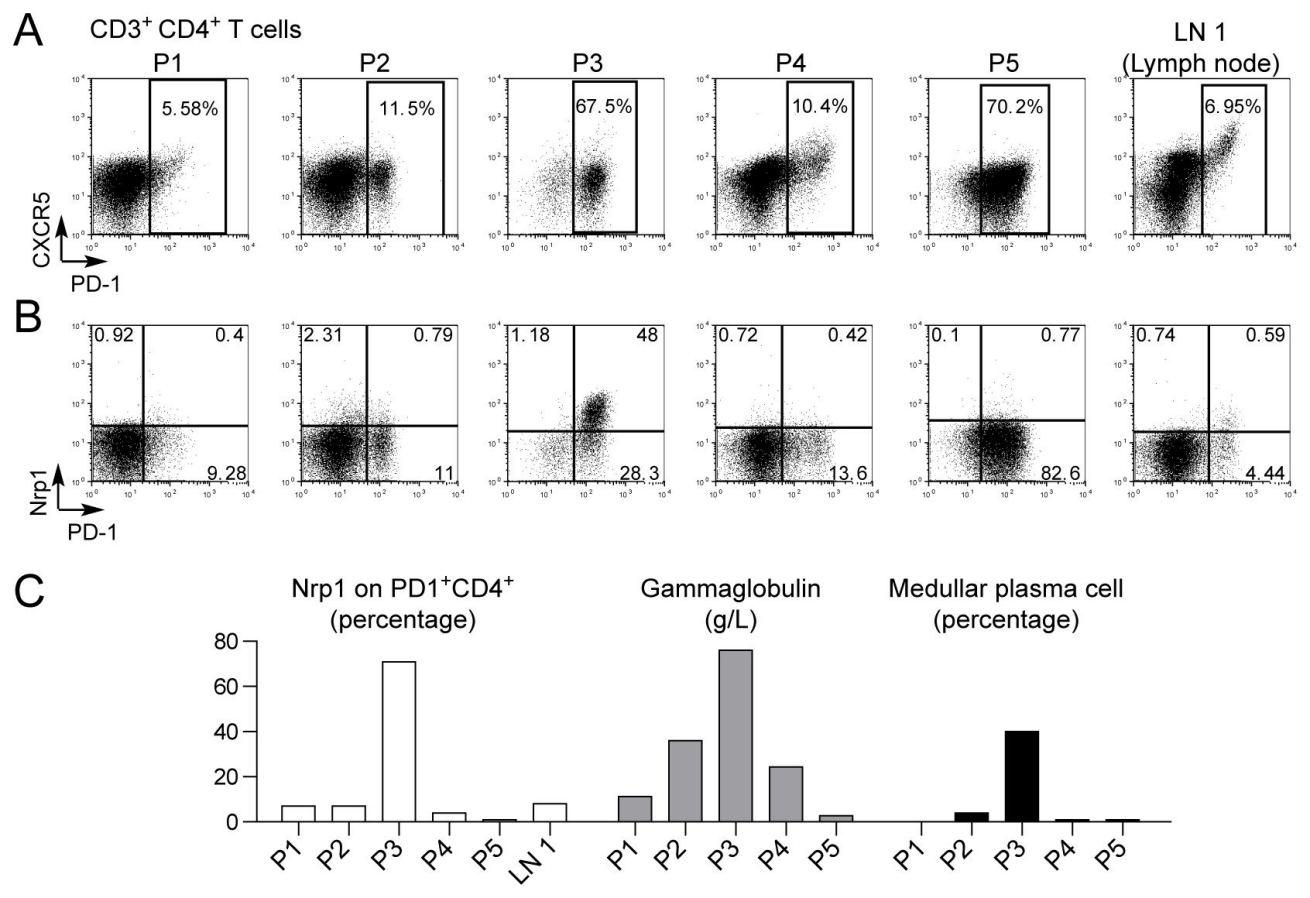
Nrp1 expression in malignant Tfh-like cells in AITL. Cell suspensions from malignant lymph node biopsies of 5 AITL patients (P1-P5) and one non-malignant reactive lymph node (LN 1) were analyzed for CXCR5 and PD-1 expression (A), and for Nrp1 and PD-1 expression (B) in gated CD3^+^ CD4^+^ T cells. (C) Bar graph display of percentage of Nrp1^+^ cells among PD1^+^ CD4^+^ T cells, serum gammaglobulin concentration and medullar plasmacytosis (percentage plasma cells among bone marrow cells) for each patient. Note that only P3 shows a significant proportion of Nrp1^+^ malignant T cells, high gammaglobulinaemia, and important medullar plasmacytosis.

Angioimmunoblastic T-cell lymphoma (AITL) is a systemic disease associated with B cell symptoms and expansion of PD-1^+^ tumoral T cells. Recently, a molecular link between AITL and Tfh cells was described [34,35], suggesting that AITL cells were transformed Tfh cells. In this study, we analyzed Nrp1 expression in homogenized lymph node biopsies of five patients (P1-P5) presenting AITL. The clinical and biological parameters of the patients’ diseases are detailed in Table 1. Briefly, all patients but one had autoimmune symptoms, they presented different ECOG status from 0 to 4 (0: fully active, able to carry on all pre-disease performance without restriction; 4: completely disabled, cannot carry on any self-care, totally confined to bed or chair [36]), three patients were hypergammaglobulinemic, and only one had a significant medullar plasmacytosis. In all cases the malignant CD4^+^ T cell population expressing PD-1 did not express CXCR5 in contrast to the non-malignant lymph node LN 1 (Figure 7 A and Table 1). Of note, malignant PD-1^+^ cells accounted for the majority of CD4^+^ T cells in P3 (67.5%) and P5 (70.2%). Furthermore, when we analyzed Nrp1 expression on CD4^+^ T cells (Figure 7 B) and on PD1^+^ CD4^+^ T cells (Figure 7 C and Table 1) we observed high levels only in P3. Interestingly, high expression of Nrp1 was only observed in the most severe case of AITL which was associated with a high ECOG score, a high level of gammaglobulin (76 g/L) as well as an important medullar and blood plasmacytosis (40% and 25% respectively) (Figure 7 C and Table 1). Thus, Nrp1 may be highly expressed by malignant Tfh-like cells in AITL, and Nrp1 expression may associate with deregulation of B cell differentiation and disease severity. 

**Table 1:** Angioimmunoblastic T-cell lymphoma (AITL) patients: clinical and biological description.

	**P1**	**P 2**	**P3**	**P4**	**P5**	**LN 1**
**B symptoms**	no	yes	yes	yes	yes	-
**Autoimmunity**	yes (antinuclear antibodies)	yes (autoimmune hemolytic anemia; smooth muscle antibodies; peripheral thrombocytopenia)	yes (cutaneous vasculitis)	yes (autoimmune hemolytic anemia; type 3 cryoglobulin)	no	-
**ECOG status**	0	2	**4**	3	1	
**Grade**	2	3-4	3-4	4	3	-
**hypergammaglobulinemia**	no	yes	yes	yes	no	-
**gamma globulin (g/L)**	11,2	36,1	**76**	24,4	2,8	-
**medullar plasmacytosis**	ND	4%	**40%**	1%	1%	-
			**(25% blood)**			
**Lymph node sample**	cervical	cervical	cervical	axillary	cervical	cervical
**% of CD4^+^ PD1^*+*^**	5,42	11,3	67,5	10,4	70,5	6,95
**CXCR5 expression**	no	no	no	yes (low)	no	yes
**% of NrP1^*+*^PD1^*+*^ on CD4**	0,4	0,8	**48**	0,42	0,83	0,59
**% of Nrp1 on PD1^*+*^CD4^+^**	7,38	7,08	**71,11**	4,04	1,18	8,49

## Discussion

Nrp1 expression in human T cells is restricted to a subset of CD4^+^ T cells in secondary lymphoid organs. Although Nrp1^+^ CD4^+^ T cells may co-express CD25 and exert regulatory function in some cancer-draining lymph nodes [24], in non-malignant lymph nodes and tonsils, Nrp1^+^ CD4^+^ T cells have a non-regulatory (CD25^-^ FoxP3^-^) memory (CD45RA^-^ CD45RO^+^) phenotype [25]. Nrp1^+^ CD4^+^ T cells also express the activation marker CD69 and the GC Tfh-related molecule CD57 [32]. In the *sanroque* mouse model of autoimmunity, massive Tfh differentiation and activity is associated with increased Nrp1 expression at the mRNA level [37]. Our study provides strong phenotypic and functional evidence that Nrp1^+^ T cells in humans are a subset of Tfh cells: Nrp1^+^ T cells express all the canonical Tfh markers (CXCR5, ICOS, PD-1), support the survival and differentiation of memory B cells *in vitro*, and have a gene expression profile characteristic of the Tfh cell lineage.

Nrp1 is induced on human peripheral blood T cells after *in vitro* activation with strong T cell receptor (TCR) crosslinking [25]. In humoral immune responses, cognate B cell / T cell interactions involving TCR / pMHCII interactions drive plasmablast differentiation, GC B cell development and high affinity B cell selection [38]. Tfh cells are thus expected to transiently express TCR-regulated activation markers during, or shortly after, their interactions with B cells. Our *in vitro* experiments show that Nrp1 expression can be induced on Tfh cells, but not on non-Tfh cells, after culture with memory B cells only. Of note, Nrp1 expression on Tfh cells is dependent on direct contact with B cells and is optimal when Tfh and B cells from the same donor are used, which argues for cognate interactions being required for Nrp1 induction. In the absence of superantigen, antigen-experienced Tfh cells should only be able to make cognate interactions with - and help - B cells with the same antigen specificity. The best Nrp1 induction on Tfh cells and B cell terminal differentiation were observed using memory B cells, which are by definition antigen-experienced, thus supporting the idea that antigen-specific interactions trigger Nrp1 induction in Tfh cells. Nrp1 expression by Tfh cells is transient, because sorted Nrp1^+^ Tfh cells lose Nrp1 expression if cultured in the absence of B cells. Collectively, our data support a model in which Nrp1 expression transiently marks only Tfh cells that make productive cognate interactions with antigen-experienced B cells. 

In the GC reaction, Tfh cell-based selection of high affinity GC B cells in the light zone induces cell cycle entry, migration to the dark zone, and terminal differentiation into plasma cells [38]. Little is known on the impact of such cognate interactions on Tfh cells. Although B cells are not required for the first stage of Tfh cell differentiation [26], cognate interactions between antigen-experienced T and B cells reinforce the molecular program of Tfh cells and are required for GC formation [8]. We show here that Nrp1^+^ Tfh cells have higher expression of IL21R but lower expression of PRDM1 (encoding Blimp-1, a potent negative regulator of Bcl6 and of Tfh cell differentiation [39]) than Nrp1^-^ Tfh cells, which suggests that B cell contact during terminal B cell differentiation also re-activates the Tfh differentiation program. Moreover, increased expression of CXCL13, IL4, PDCD1(encoding PD-1), TNFSFR9 (encoding CD137 or 4-1BB) and TNFSFR18 (encoding GITR), as observed in Nrp1^+^ Tfh cells, may be a signature of cognate B cell contact in Tfh cells. In mice, GC Tfh cells express higher levels of PD-1 and are the main producers of IL-4 [40]. In human, GC Tfh cells produce higher levels of CXCL13 and IL-4, but much less IL-10 than non-GC Tfh cells [41]. Our gene expression analyses show increased expression of IL4, PDCD1 and CXCL13, but reduced expression of IL10, in Nrp1^+^ Tfh cells compared to their Nrp1^-^ counterparts. These results suggest that cognate interactions with B cells favor GC Tfh cells differentiation and maintenance in humans. “Preferential” expression of Nrp1 by CD57^+^ Tfh cells suggests that the phenomenon we describe is mostly concentrated to the GC. However, Nrp1 is also observed on CXCR5^+^ CD57^-^, which implicates that Nrp1 expression is not specific to a subset of Tfh cells, but rather characteristic of an activation state that is induced during, and shortly after, interaction with cognate B cells inside and outside the GC.

Nrp1 functions as a homotypic adhesion molecule in immunological synapses involving dendritic cells and CD4^+^ T cells in human [17] or Treg cells in mice [20]. The absence of Nrp1 expression in B cells, *ex vivo* or after culture with T cells, makes it unlikely that Nrp1 serves this function in human GCs. In the nervous system, Nrp1 serves as a coreceptor, along with plexin family members, for secreted chemorepellent class 3 semaphorins [14]. In the thymus, Nrp1 and one of its ligands, semaphorin 3A, interact to control the migration of thymocytes along thymic epithelial cells [42,43]. In mice, GC B cells express axon guidance molecules like plexin B2 [44]. Therefore, it is likely that axon guidance cues and their receptors play a role in the migration of cells in the GC environment. Nrp1 expression in Tfh cells may be necessary for their migration to and/or retention in the GC.

AITL is a peripheral T cell lymphoma that derives from Tfh cells [34,35,45]. Our study of five cases of AITL revealed that Nrp1 expression in malignant PD-1^+^ T cells is not systematic. Only one case had high Nrp1 expression on malignant Tfh-like cells, which was associated with increased gammaglobulinaemia and massive blood and medullary plasmacytosis. This suggests that Nrp1 expression in AITL may be a correlate of increased Tfh-like activity leading to severe B cell symptoms. Nrp1 is expressed in numerous cancer types and is usually associated with a bad prognosis due to its role in tumor angiogenesis and migration [46]. Monoclonal antibodies [47] and small peptides [48,49] that block Nrp1-mediated functions are being developed as cancer therapeutics. Other strategies that use Nrp1 as an endocytic receptor to target drugs specifically to Nrp1^+^ cells in the tumor microenvironment also show great promise [50,51]. Such strategies may prove efficient to target Nrp1^+^ Tfh cells in the treatment of severe AITL.

## Supporting Information

Figure S1 **Gating strategy used for Tfh and B cell populations analysis in human tonsils and lymph nodes.** Representative gating strategy in human tonsil sample. Doublets are excluded from the lymphocyte gate. CD3^+^ CD4^+^ T cells are analyzed for PD-1, CXCR5, ICOS and Nrp1 expression for identification of non-Tfh and Tfh subsets. B cells (CD3^-^ CD19^+^) are divided into naive B cells (IgD^+^ CD38^-^), GC B cells (IgD^-^ CD38^+^), memory B cells (IgD^-^ CD38^-^), and plasmablasts (IgD^-^ CD38^hi^).
(TIF)

Figure S2 **Circulating Tfh cells do not express Nrp1.** Representative gating used for analyzing Nrp1 expression in circulating CD3^+^ CD4^+^ CXCR5^+^ Tfh cells (n=3). Numbers represent the mean ± SD percentage of CD3^+^ CD4^+^ T cells in the corresponding quadrant. Note the absence of Nrp1 expression on CXCR5^+^ circulating Tfh cells.
(TIF)

Table S1 Gene expression of Nrp1^-^ and Nrp1^+^ Tfh cells.
(XLSX)
