# Supplementary figures and images for: Neuropilin-1 Expression Characterizes T Follicular Helper (Tfh) Cells Activated during B Cell Differentiation in Human Secondary Lymphoid Organs
Source: PLoS One. 2013 Dec 30;8(12):e85589. doi: 10.1371/journal.pone.0085589 (PMC3875584; doi:10.1371/journal.pone.0085589)

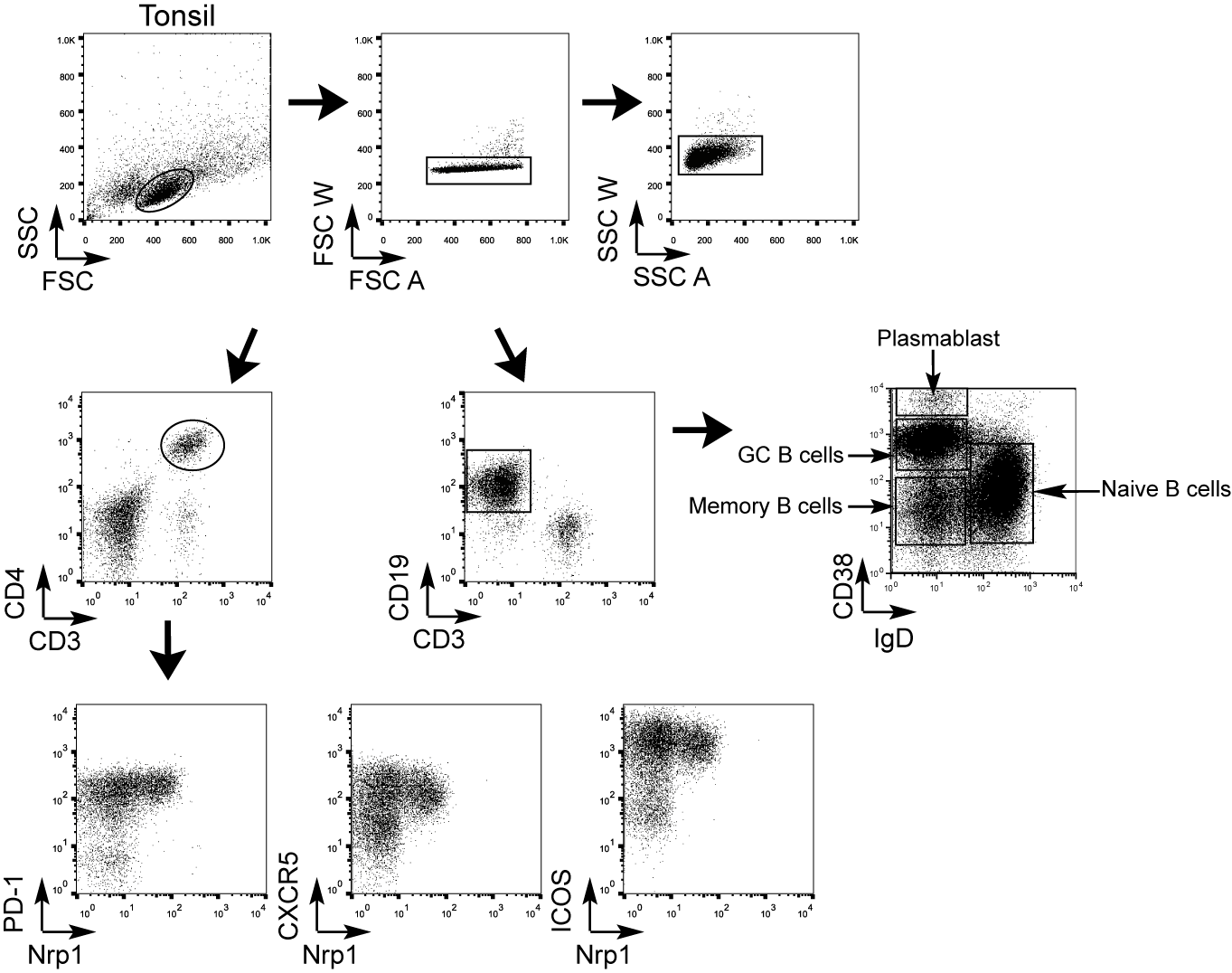

Supplement: Figure S1 — Gating strategy used for Tfh and B cell populations analysis in human tonsils and lymph nodes. Representative gating strategy in human tonsil sample. Doublets are excluded from the lymphocyte gate. CD3+ CD4+ T cells are analyzed for PD-1, CXCR5, ICOS and Nrp1 expression for identification of non-Tfh and Tfh subsets. B cells (CD3- CD19+) are divided into naive B cells (IgD+ CD38-), GC B cells (IgD- CD38+), memory B cells (IgD- CD38-), and plasmablasts (IgD- CD38hi). (TIF) [file pone.0085589.s001.tif]

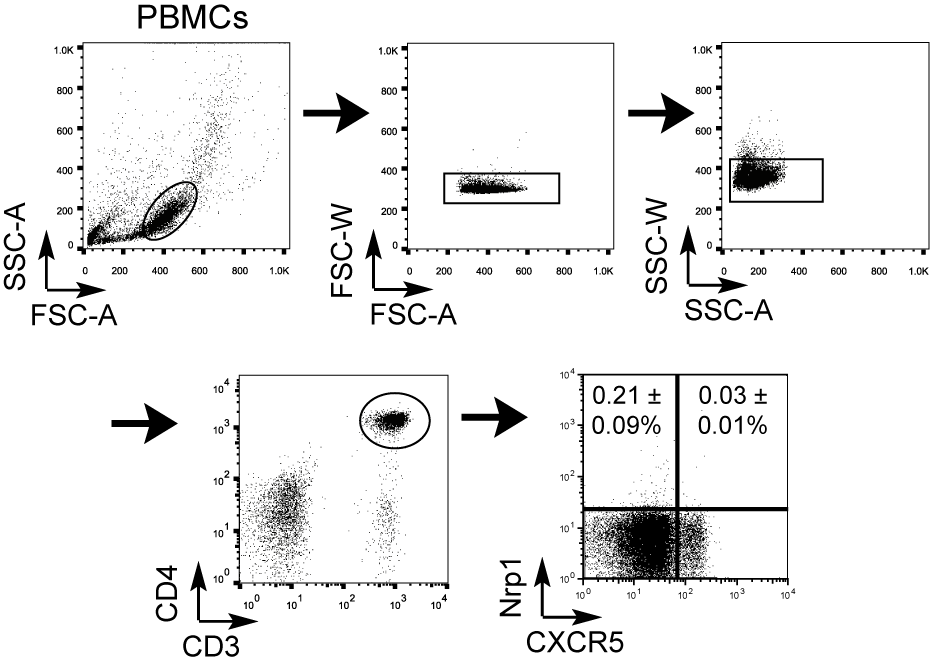

Supplement: Figure S2 — Circulating Tfh cells do not express Nrp1. Representative gating used for analyzing Nrp1 expression in circulating CD3+ CD4+ CXCR5+ Tfh cells (n=3). Numbers represent the mean ± SD percentage of CD3+ CD4+ T cells in the corresponding quadrant. Note the absence of Nrp1 expression on CXCR5+ circulating Tfh cells. (TIF) [file pone.0085589.s002.tif]
